# Comprehensive analysis of fatty acid desaturase 3 in clear cell renal cell carcinoma: insights into tumor progression, immune microenvironment, and clinical outcomes

**DOI:** 10.3389/fimmu.2026.1817492

**Published:** 2026-05-14

**Authors:** Si-Tao Chen, Duan-Rui Zhou, Yun-Jun Ge, Lei Chang, Shu-Hui Guo, Guo-Sheng Wu, Ning-Han Feng

**Affiliations:** 1 Department of Urology, Jiangnan University Medical Center, Wuxi, China; 2 Wuxi School of Medicine, Jiangnan University, Wuxi, China; 3 MOE Medical Basic Research Innovation Center for Gut Microbiota and Chronic Diseases, Wuxi School of Medicine, Jiangnan University, Wuxi, China; 4 School of Health and Medicine, Wuxi Taihu University, Wuxi, China

**Keywords:** clear cell renal cell carcinoma, fatty acid desaturase 3, lipid metabolism, tumor immune microenvironment, tumor progression and prognosis

## Abstract

**Background:**

Clear cell renal cell carcinoma (ccRCC or KIRC) is a malignant neoplasm characterized by reprogrammed lipid metabolism. Fatty acid desaturase 3 (FADS3), a sphingoid Δ14Z desaturase, is required for the synthesis of unsaturated fatty acids in tumor biology. However, the role of FADS3 in ccRCC progression and prognosis and in modulating the tumor immune microenvironment (TIME) remains to be elucidated.

**Methods:**

The ccRCC transcriptomic datasets were obtained from the TCGA, GEO, and GTEx databases. Mendelian randomization (MR) and single-cell RNA sequencing analyses were used to investigate the associations of FADS3 with lipid metabolism, and differential expression genes in ccRCC. Bioinformatics analysis was also used to investigate the association of FADS3 expression with tumor progression, prognosis, TIME, and potential pathogenic mechanism in ccRCC. FADS3 expression in ccRCC cell lines was confirmed by qRT-PCR, western blotting, RNA-Seq. FADS3 roles in ccRCC were assessed by functional assays including cell proliferation, migration, invasion, and colony formation using wild-type and FADS3-knockdown cell lines.

**Results:**

Lipid metabolism was found to be upregulated in ccRCC tumor tissues based on comprehensive bioinformatic analysis. FADS3 was among the upregulated genes associated with lipid metabolism, which was expressed not only in ccRCC tumor cells but also in cells of the TIME, such as tumor-associated macrophages (TAMs). High FADS3 expression remodeled the TIME and predicted poor prognosis in ccRCC. Functional assays demonstrated that FADS3 knockdown markedly suppressed ccRCC cell proliferation, migration and invasion. Transcriptomic analyses further suggested that FADS3 may promote ccRCC progression through activation of oncogenic and metabolic signaling pathways, such as the PI3K/Akt pathway.

**Conclusion:**

FADS3 is a lipid metabolism-associated oncogenic driver in ccRCC, and its upregulation remodels the TIME and predicts poor prognosis. FADS3 may represent a potential therapeutic target for ccRCC treatment.

## 1 Introduction

Renal cell carcinoma (RCC) accounts for approximately 2% of all malignancies worldwide, among which ccRCC is the predominant histological subtype (1–4). According to recent global and population-based studies, the incidence of ccRCC has shown a steady increase over the past decades (5, 6). Compared to other solid tumors, ccRCC progression is highly dependent on lipid metabolism (7–9). Inactivation of tumor suppressor genes and activation of hypoxia-inducible factors drive enhanced lipid uptake, upregulation of fatty acid synthesis, and accumulation of lipid droplets in ccRCC (10, 11). The lipid profile of ccRCC tissues differs significantly from that of normal renal tissue, involving comprehensive dysregulation of metabolic pathways related to cholesterol, free fatty acids (FFAs), and polyunsaturated fatty acids (PUFAs) (12, 13). Multiple key enzymes involved in fatty acid synthesis and desaturation, such as FASN, SCD1, and ACLY, promote the growth, survival, and drug resistance of ccRCC (14–17). ccRCC is increasingly regarded as a metabolic disorder driven by lipid metabolic reprogramming, rather than merely a conventional solid tumor (9). However, the regulatory network of lipid metabolism in ccRCC remains incompletely understood, and numerous potential key regulators await discovery.

Immune checkpoint inhibitor (ICI)-based combination regimens are now established as first-line therapy for advanced ccRCC according to the ESMO Clinical Practice Guidelines (18). Clinical trials have demonstrated that the combination of anti-programmed death protein 1 (PD-1) antibody pembrolizumab and vascular endothelial growth factor receptor inhibitor axitinib significantly improves overall survival, progression-free survival, and objective response rates in this setting (19). Nevertheless, a substantial subset of patients exhibits intrinsic or acquired resistance to ICIs (20). Response in ccRCC is not solely dictated by the degree of T-cell infiltration; rather, it is shaped by complex interactions among immune checkpoints, immunosuppressive cytokine networks, and tumor-intrinsic regulatory programs (21). Among these intrinsic programs, alterations in fatty acid metabolism have emerged as key modulators of immunotherapy responsiveness (22). Furthermore, dysregulated lipid accumulation and its crosstalk with ferroptosis pathways contribute to immune evasion and therapeutic resistance, highlighting actionable metabolic vulnerabilities in this disease (23). Consequently, targeting lipid metabolism represents a promising combination strategy to overcome the limitations of current immunotherapy for ccRCC treatment.

FADS3 belongs to the FADS gene family and, like FADS1/2, is a key enzyme involved in fatty acid desaturation during lipid metabolism (24). However, its specific substrate spectrum and catalytic sites differ significantly from FADS1/2 (25, 26). Recent studies have shown that FADS3 possesses Δ14Z desaturase activity on the sphingoid base, thereby altering cellular and plasma sphingolipid profiles (27, 28). The sphingolipid family (ceramides, sphingosine-1-phosphate, etc.) plays a dual role in apoptosis, cell proliferation, migration, and immune regulation (29, 30). Alterations in enzymatic mechanisms (e.g., desaturases, ceramide synthases, sphingosine kinases) are considered to promote or inhibit tumor progression and represent potential therapeutic targets (30, 31). The changes in sphingoid base types influenced by FADS3 can theoretically affect tumor cell fate by altering the balance of these signaling lipids (30). Multiple omics and bioinformatics studies in several solid tumors suggest that FADS3 may promote tumor progression (32, 33). However, the biological and clinical significance of FADS3 in ccRCC remains largely unexplored.

Herein, we demonstrate that FADS3 is upregulated in ccRCC, remodels the TIME, and serves as a predictor of poor prognosis. Functional assays reveal that FADS3 knockdown markedly inhibits ccRCC cell proliferation, migration, and invasion. Mechanistically, FADS3 may promote ccRCC progression through activation of the PI3K/Akt signaling pathway. These findings identify FADS3 as a potential metabolic signaling-related target for precision therapy in ccRCC.

## 2 Materials and methods

### 2.1 Data source

Four ccRCC transcriptome datasets were obtained from the TCGA and GEO databases for differential analysis, while clinical data of ccRCC were obtained from the TCGA. The information of the four datasets is shown in **Table 1**. High-density lipoprotein (HDL) (ebi-a-GCST90018956), low-density lipoprotein (LDL) (ieu-b-5089), total cholesterol (TC) (ebi-a-GCST90018974), triglycerides (TG) (ebi-a-GCST90018975), monounsaturated fatty acids (MUFA) (ebi-a-GCST90092928), PUFA (ebi-a-GCST90092939), saturated fatty acids (SFA) (ebi-a-GCST90092980), and trans fatty acids (TFA) (ebi-a-GCST90092987) were obtained from IEU database (https://opengwas.io/datasets/). The GWAS data of ccRCC (finngen_R10_C3_KIDNEY_CLEAR_CELL_CARCINOMA_EXALLC) were obtained from Finngen database (https://www.finngen.fi/en). Lipid metabolism related data sets were derived from Lipid Map (https://www.lipidmaps.org) and Genecard (https://www.genecards.org). eQTL data for genes were obtained from GTEx database (https://www.gtexportal.org/home/).

**Table 1:** The data source of ccRCC transcriptome datasets.

Data	nControl	Source
TCGA-KIRC	72	https://portal.gdc.cancer.gov
GSE36895	23	https://www.ncbi.nlm.nih.gov/geo/query/acc.cgi?acc=GSE36895
GSE66272	27	https://www.ncbi.nlm.nih.gov/geo/query/acc.cgi?acc=GSE66272
GSE126964	11	https://www.ncbi.nlm.nih.gov/geo/query/acc.cgi?acc=GSE126964

### 2.2 Weighted gene co-expression network analysis

In order to map the key genes of ccRCC comprehensively, we performed WGCNA on the intersection genes of the four ccRCC data sets considering the mutual correlations and associations between gene sets and phenotypes in the normal and tumor groups. A scale-free co-expression network was constructed using the WGCNA package in R software, with a soft threshold power of β = 18 set to identify key modules. Modules were identified using Pearson’s correlation test, with *P*-value < 0.05 as the threshold for significance.

### 2.3 MR analysis

MR analysis was performed to verify the potential association between lipid metabolism and ccRCC, and subsequently to validate the selected ccRCC core genes related to lipid metabolism using GTEx whole blood-derived eQTL data. Three core assumptions of MR were strictly followed in this study: 1) Instrumental Variables (IVs) were strongly correlated with exposure factors (correlation hypothesis); 2) the instrumental variables were independent of confounders for the exposure-outcome relationship (independence assumption); 3) Instrumental variables affect outcomes only through exposure (excluding limiting hypotheses). Analyses were performed with the use of R software (version 4.3.0) and the “TwoSampleMR” R package (version 0.5.7).

### 2.4 Single-cell transcriptome analysis

Single-cell RNA-sequencing data were obtained from the GEO database, including GSE156632 and GSE159115. GSE156632 contains ccRCC tumor and matched adjacent normal samples, whereas GSE159115 includes renal tumor and benign kidney tissue samples; only ccRCC and benign kidney samples were retained for analysis. Data processing was performed using the Seurat package in R. After quality control, the data were normalized, highly variable genes were identified, and principal component analysis was conducted. Harmony was applied for dataset integration and batch-effect correction, followed by clustering and UMAP visualization. Cell types were annotated according to canonical marker genes and grouped into six major populations: epithelial, immune, myeloid, endothelial, fibroblast, and smooth muscle cells. Because ccRCC is of epithelial origin, subsequent analyses focused on the epithelial compartment. Epithelial cells were extracted for re-clustering, and copy number variation profiles were inferred using the inferCNV package based on raw counts and genomic gene-order information. Non-malignant cell populations were used as reference cells, whereas putative tumor epithelial cells were defined as the observation group. Based on inferCNV-derived CNV patterns, epithelial cells were classified into high-confidence malignant and non-malignant populations, and clusters with mixed CNV signals were excluded. Differential expression analysis between malignant and non-malignant epithelial cells was performed using the FindMarkers function in Seurat, and results were visualized according to log2 fold change and the difference in the proportion of expressing cells, with FADS3 highlighted as a candidate gene.

### 2.5 RNA and protein expression analysis

To validate the results from the TCGA database above, we also acquired and used expression microarray data from GSE46699 (containing 60 tumor samples and 56 normal samples) and GSE53757 (containing 72 tumor samples and 72 normal samples) from the Gene Expression Omnibus (GEO) database (http://www.ncbi.nlm.nih.gov/projects/geo/). The Wilcoxon rank-sum test was used to determine significance levels (*P*-value < 0.05 indicated statistical significance) to confirm the expression level of FADS3 in ccRCC. Concurrently, we utilized the UALCAN database to analyze the difference in FADS3 protein expression between adjacent normal tissues and malignant tissues in ccRCC online.

### 2.6 Clinical staging and pathological grading

We assessed the relationship between FADS3 mRNA expression and various clinical characteristics of ccRCC, including clinical T stage, N stage, metastasis status, and tumor stage grouping. RNA sequencing expression profiles and corresponding clinical and pathological information for ccRCC (542 tumor samples) were downloaded from the TCGA dataset. Statistical analysis and ggplot2 plotting (v3.3.2) were completed using R program v4.0.3, *P*-value < 0.05 was considered statistically significant.

### 2.7 Survival analysis and univariate and multivariate Cox regression analysis

Tumor samples from the TCGA-KIRC cohort were divided into high-expression and low-expression groups using the median FADS3 expression level as the cutoff value. Kaplan-Meier (KM) survival analysis was used to evaluate the relationship between the FADS3 high-expression and low-expression groups and Overall Survival (OS), Progression-Free Interval (PFI), and Progression-Free Survival (PFS), with a P-value threshold of < 0.05.To further investigate the relationship between FADS3 and overall survival (OS), we performed univariate and multivariate Cox regression analyses to determine whether FADS3 and other clinical characteristics (age, gender, total tumor stage, pathological T stage, N stage, M stage) could serve as independent factors associated with OS in ccRCC patients in the TCGA database (*P*-value threshold < 0.05). Multivariate Cox regression was used to determine the independent effects and interactive effects of FADS3 expression and clinical variables on the prognosis of ccRCC patients (*P*-value threshold < 0.05).

### 2.8 PPI network construction and hub gene identification

Spearman correlation tests were used to analyze the correlation between various genes and FADS3. Genes were ranked according to correlation coefficients for those with an adjusted *P*-value < 0.05. The top 50 genes positively and negatively correlated with FADS3 were selected for subsequent analysis. We established a Protein-Protein Interaction (PPI) network based on the aforementioned 100 genes using the STRING database (https://string-db.org/). Subsequently, the network was visualized using Cytoscape software, and the top 10 hub genes were identified using the CytoHubba plugin. Kaplan-Meier plots for these 10 hub genes were generated as previously described.

### 2.9 Construction and validation of the ccRCC prognostic model

Gene expression profiles (HTSeq-FPKM or TPM, Level 3) and corresponding clinical information for ccRCC were retrieved from the TCGA database, comprising 542 KIRC tumor tissues and 72 adjacent non-tumor kidney tissues for subsequent differential expression and prognostic analyses. To identify key genes associated with overall survival, univariate Cox regression was initially performed with a significance threshold of *p* < 0.05, and the resulting candidate prognostic factors were subjected to Least Absolute Shrinkage and Selection Operator (LASSO) regression using the “glmnet” R package to enhance model stability and prevent overfitting. The remaining genes were subsequently incorporated into a multivariate Cox proportional hazards model to determine independent prognostic factors, and the final risk score was calculated using the formula: Risk Score = (FADS3 Expression × 0.0368) − (DLAT Expression × 0.0462) + (TCIRG1 Expression × 0.0323). Patients were then stratified into high-risk and low-risk groups based on the median risk score threshold, with model performance for 1-, 3-, and 5-year overall survival evaluated using time-dependent Receiver Operating Characteristic (ROC) curves generated via the “survivalROC” R package and Kaplan-Meier survival curves constructed using the “survival” R package.

To evaluate clinical applicability, both univariate and multivariate Cox regression analyses were performed integrating standard clinicopathological characteristics and the calculated risk score; a nomogram was constructed using the “rms” R package to predict the probability of 1-, 3-, and 5-year overall survival based on significant variables, and internally validated using Bootstrap resampling (1,000 iterations) with predictive accuracy and calibration assessed using the Area Under the Curve (AUC) and calibration plots, respectively. Additionally, a Random Forest machine-learning classifier was employed to identify key diagnostic features distinguishing tumor from normal tissues, trained using the “randomForest” R package with 500 decision trees and the random seed fixed to 1234 for reproducibility, where feature importance scores were calculated based on the reduction in node impurity with higher values indicating greater contribution to diagnostic classification.

### 2.10 Analysis of immune infiltration and immune checkpoints

The “Estimate” package in R software (v4.2.1) was used to assess the Stromal Score, Immune Score, and ESTIMATE Score of the ccRCC microenvironment, grouped by high and low FADS3 expression. Using the same grouping method, the CIBERSORT method (based on linear support vector regression principles) was employed to provide expression data for 22 common immune infiltrating cells, including different cell types and functional states. The abundance of immune cells was estimated by deconvolving the expression matrix of immune cell subtypes. Additionally, the Single Sample Gene Set Enrichment Analysis (ssGSEA) method was used to calculate the enrichment score of each immune cell in tumor samples based on immune cell marker genes, analyzing the immune infiltration of 28 immune cells in ccRCC. The Wilcoxon rank-sum test was used to compare and visualize the immune cell enrichment scores between the FADS3 high-expression and low-expression groups. Furthermore, we utilized the CIBERSORT and ssGSEA results to perform Spearman analysis to obtain correlations between FADS3 and various immune cells and immune-regulatory genes. By obtaining the gene list files for immune checkpoints and the previously obtained gene expression matrix from TCGA, we analyzed the correlation between FADS3 and immune checkpoints using R software algorithms such as limma and reshape2 (*P*-value < 0.05 was considered statistically significant).

### 2.11 Prediction of immunotherapy response

Based on TCGA-KIRC transcriptome data grouped by high and low FADS3 expression, the Tumor Immune Dysfunction and Exclusion (TIDE; http://tide.dfci.harvard.edu/) method was used to evaluate the potential clinical efficacy of immunotherapy in different FADS3 expression groups. Significant differences were statistically compared using the Wilcoxon rank-sum test. The TIDE tool provides an in-depth analysis of immune cell infiltration and functional status in the tumor microenvironment, helping to assess immune cell composition and its potential impact on tumor growth and treatment response; a higher TIDE score indicates a greater likelihood of immune escape, suggesting a lower likelihood of patient benefit from immunotherapy. Concurrently, we obtained immunogenomic analysis results for TCGA-KIRC, such as the Immunophenoscore (IPS), from the TCIA database. Differences in four types of IPS were calculated using R software, and significant differences were statistically compared using the Wilcoxon rank-sum test.

### 2.12 Functional and pathway enrichment analysis

To further understand the biological processes and signaling pathways associated with FADS3 gene expression, we initially used the “Limma” package to evaluate mRNA expression differences between the FADS3-high and FADS3-low subgroups. A *P*-value < 0.05 and logFC ≥ 1 were considered significant. Subsequently, utilizing TCGA ccRCC transcriptome data, we performed Kyoto Encyclopedia of Genes and Genomes (KEGG) pathway enrichment analysis and Gene Ontology (GO) enrichment analysis. To enrich KEGG pathways and detect GO functions of differential genes between FADS3-high and FADS3-low groups, we used the “ClusterProfiler” package. A pathway was considered enriched if *P*-value < 0.05 or FDR < 0.05.

### 2.13 Immunofluorescence staining of tumor tissues

Multiplex immunofluorescence staining was performed on a ccRCC tissue microarray containing tumor and adjacent normal tissues. The tissue microarray (catalog no. HKidE150CS03) was obtained from Shanghai Outdo Biotech Co., Ltd. (Shanghai, China), with ethical approval number SHYJS-CP-1904005. Following antigen retrieval and blocking, slides were incubated overnight at 4 °C with primary antibodies against FADS3 (Proteintech, 15205-1-AP) and CD11b (Proteintech, 66519-1-Ig). Subsequently, sections were incubated with fluorescent secondary antibodies (Alexa Fluor 568-conjugated anti-mouse IgG, sms1AF568-1; Alexa Fluor 488-conjugated anti-rabbit IgG, srbAF488-1) and counterstained with DAPI in the dark. Images were acquired under identical settings, and fluorescence intensities were quantified using ImageJ.

### 2.14 Cell culture and lentiviral knockdown

Two renal cancer cell lines, 786-O and Caki-1, were obtained from the National Collection of Authenticated Cell Cultures (Shanghai, China). Both cell lines were cultured in RPMI-1640 medium (Sigma, St. Louis, MO, USA) supplemented with 10% fetal bovine serum (HyClone, Logan, Utah, USA), 100 U/ml penicillin, and 100 μg/ml streptomycin. Cells were maintained in a humidified incubator at 37 °C with 5% CO2.

To construct stable FADS3 knockdown cell lines, 786-O and Caki-1 cells were seeded at 30-40% confluency one day prior to infection. Cells were infected with commercially purchased FADS3 shRNA lentiviral particles (carrying puromycin resistance and a Green Fluorescent Protein (GFP) reporter gene) at a multiplicity of infection (MOI) of 10, with the addition of 8 μg/mL Polybrene to enhance infection efficiency. The shRNA sequences were as follows: shRNA1-F, 5’-gatcgCCTTCCATCAAGATCTCAATTctcgagAATTGAGATCTTGATGGAAGGTTTTTT-3’; shRNA2-F, 5’-gatcgGGCAAGAAGAAACGCAGATACctcgagGTATCTGCGTTTCTTCTTGCCTTTTTT-3’; shRNA3-F, 5’-gatcgGTGCATGCAGTGGGCGGATTTctcgagAAATCCGCCCACTGCATGCACTTTTTT-3’. The medium was replaced with fresh complete medium 24 hours after infection. After 48 hours of infection, puromycin selection was initiated (Caki-1: 2 μg/mL; 786-O: 1.5 μg/mL) and maintained for 5–7 days until uninfected cells completely died. Transduction efficiency was analyzed using fluorescence microscopy. Stable cell lines were analyzed for FADS3 expression levels using quantitative real-time PCR (qRT-PCR) and Western blot to verify knockdown efficiency.

### 2.15 RT-qPCR and western blot

Total RNA was extracted using TsingZol Total RNA extraction reagent (TSINGKE, TSP401). cDNA was synthesized using 5× All-In-One RT Master Mix (Applied Biological Materials, G490) according to the manufacturer’s protocol. RT-qPCR was performed using PowerUp™ SYBR™ Green Master Mix (Applied Biosystems™, A25742) and a PIKOREAL 96 Real-Time PCR System (ThermoFisher). β-actin served as the internal control, and the relative gene expression levels were quantified using the 2^-ΔΔCT method. The amplification primer sequences were as follows: FADS3-F-GGCTCAGTCCTGGTGTCTGC , FADS3-R-GTGCTGGAAGTGGCGGAAGT, β-actin-F-GAGGTGAGGTGGGTCC, β-actin-R-GAAGAGGTGGGTTTTC. Lentivirus-transfected 786-O and Caki-1 cells were lysed using cell lysis buffer containing protease inhibitors (Beyotime, Shanghai). Protein concentration was determined using a BCA Protein Assay Kit (BIO-RAD, CA, USA). Protein samples were loaded onto SDS-PAGE for electrophoresis and transferred to PVDF membranes (BIO-RAD, CA, USA). After blocking with 5% non-fat milk, membranes were treated with primary antibody (1:1000 dilution) and secondary antibody (1:3000 dilution). The FADS3 antibody was obtained from Proteintech Group, Inc (Wuhan, China), and all others were purchased from Cell Signaling Technology (Beverly, MA, USA). Finally, protein membranes were treated with enhanced chemiluminescence detection buffer, and protein bands were captured using a ChemiDOC MP system (Bio Rad Laboratories; Hercules, CA, USA).

### 2.16 Cell proliferation assays and colony formation assay

Cell proliferation was analyzed with Cell Counting Kit-8 (CCK-8) or 3-(4,5-dimethylthiazol-2-yl)-2,5-diphenyl-2H-tetrazolium bromide (MTT) assay. Logarithmic growth phase cells were prepared into a cell suspension of 2×10^4 cells/ml. 100 µL was added to each well of a 96-well plate, with 3 replicate wells for each cell type (2×10^3 cells/well), using 100 µl of culture medium as a blank control. Plates were incubated overnight at 37 °C. After treating cells for 0, 12, 24, 36, and 48 hours respectively, Cell Counting Kit-8 (CCK-8) mixed with serum-free essential medium at a 1:10 volume ratio (100 µL per well) was added to the test wells. Cells were incubated in a 37 °C, 5% CO2 incubator for 1 hour; absorbance at 450 nm was measured using a microplate reader. For MTT assay, 20 µL of MTT solution (5 mg/mL) was added to each well and incubated for 4 h at 37 °C. The medium was then removed, and 150 µL of DMSO was added to dissolve the formazan crystals. Absorbance was measured at 570 nm using a microplate reader.

Logarithmic growth phase cells were digested with 0.25% trypsin to prepare a single-cell suspension. The cell suspension was serially diluted, and cells were seeded into culture dishes at an appropriate density. Dishes were gently rotated to disperse cells evenly and incubated at 37 °C, 5% CO2, and saturated humidity for 2–3 weeks, with medium changes every 5 days. Culture was terminated when macroscopic clones appeared. Supernatant was discarded, and cells were carefully washed twice with PBS. Cells were fixed with 4% paraformaldehyde for 15 minutes, then stained with crystal violet solution for 5–10 minutes. After washing with running water and air drying, plates were photographed, and clones were counted visually. Clone formation rate = (number of clones/number of inoculated cells) × 100%.

### 2.17 Scratch wound healing assay and transwell assay

After digestion and counting, 5×10^5 cells from each group were added to a six-well plate and cultured overnight until the bottom was fully confluent. The culture medium was aspirated, and a scratch was made using a 200 μL pipette tip. Cells were washed with PBS to remove debris, and basal medium was added for continued culture. Photos were taken at 0h and 24h, and the scratch area at different time points was analyzed using ImageJ software.

24-well Transwell chambers with 8 µm membrane filters (Corning) were pre-coated with Matrigel (Yesen Biotechnology Co., Ltd, Shanghai, China). Cells were seeded in the upper chamber in serum-free medium, while the lower chamber was filled with complete medium containing 10% FBS. After incubation at 37 °C for 24 hours, cells on the lower surface were fixed with 4% paraformaldehyde for 10 minutes and stained with 0.1% crystal violet for 10 minutes at room temperature. Stained cells were photographed and quantified.

### 2.18 RNA sequencing and differential expression analysis

To investigate the transcriptomic alterations induced by FADS3 knockdown, RNA sequencing (RNA-seq) was performed in two ccRCC cell lines. Stable FADS3-silenced 786-O and Caki-1 cells (shFADS3) and their matched negative control cells (shNC) were prepared in biological triplicates and submitted to LC-Bio Technology Co.Ltd. (Hangzhou, China) for library construction and sequencing. Total RNA was extracted using TRIzol reagent (Invitrogen, USA) following the manufacturer’s protocol. RNA concentration and purity were measured using a NanoDrop 2000 spectrophotometer (Thermo Fisher Scientific), and RNA integrity was assessed using an Agilent 2100 Bioanalyzer. Samples with an RNA Integrity Number (RIN) ≥ 7.0 were considered qualified and used for library preparation.

Gene-level read counts were obtained using featureCounts. Differential expression analysis between shFADS3 and shNC groups was conducted using the DESeq2 package in R. Genes meeting the following criteria were defined as differentially expressed genes (DEGs): |log2 fold change| ≥ 1 and adjusted *P*-value (FDR) < 0.05.

### 2.19 Functional enrichment analysis

Functional enrichment analyses, including Gene Ontology (GO) and Kyoto Encyclopedia of Genes and Genomes (KEGG) pathway analysis, were performed on the DEGs identified in the RNA-sequencing study. We used the ClusterProfiler R package (version 4.8.3) for all enrichment calculations. To control the false discovery rate, all results were corrected using the Benjamini–Hochberg (BH) method. Pathways or GO terms were considered significantly enriched if the *P*-value < 0.05. This comprehensive analysis provided insight into the transcriptomic reprogramming after FADS3 knockdown in ccRCC cells.

### 2.20 THP-1 conditioned medium stimulation assay

THP-1 cells were treated with phorbol 12-myristate 13-acetate (PMA) to induce macrophage differentiation. Conditioned medium (CM) from FADS3-knockdown or control ccRCC cells (786-O or Caki-1) was applied to PMA-differentiated THP-1 cells for stimulation. Following incubation, total RNA was extracted, and TNF-α mRNA levels were quantified by quantitative real-time PCR (qRT-PCR). Relative TNF-α expression was normalized to the internal reference gene and calculated using the 2^-ΔΔCt method. The amplification primer sequences were as follows: hu-TNFa-F-CCTCTCTCTAATCAGCCCTCTG, hu-TNFa-R-GAGGACCTGGGAGTAGATGAG, β-actin-F-GAGGTGAGGTGGGTCC, β-actin-R-GAAGAGGTGGGTTTTC.

### 2.21 Statistical analysis

Experiments were independently repeated at least three times, and results are presented as mean ± standard deviation (SD). Differences between two groups were evaluated with Student’s *t*-test; comparisons among multiple groups were assessed by one- or two-way analysis of variance (ANOVA) as appropriate. Data were analyzed using GraphPad Prism 7.0 (GraphPad Software; San Diego, CA, USA). *P*-value < 0.05 was considered statistically significant.

## 3 Results

### 3.1 FADS3 is the core lipid metabolism-related gene in ccRCC

**Figure 1:**
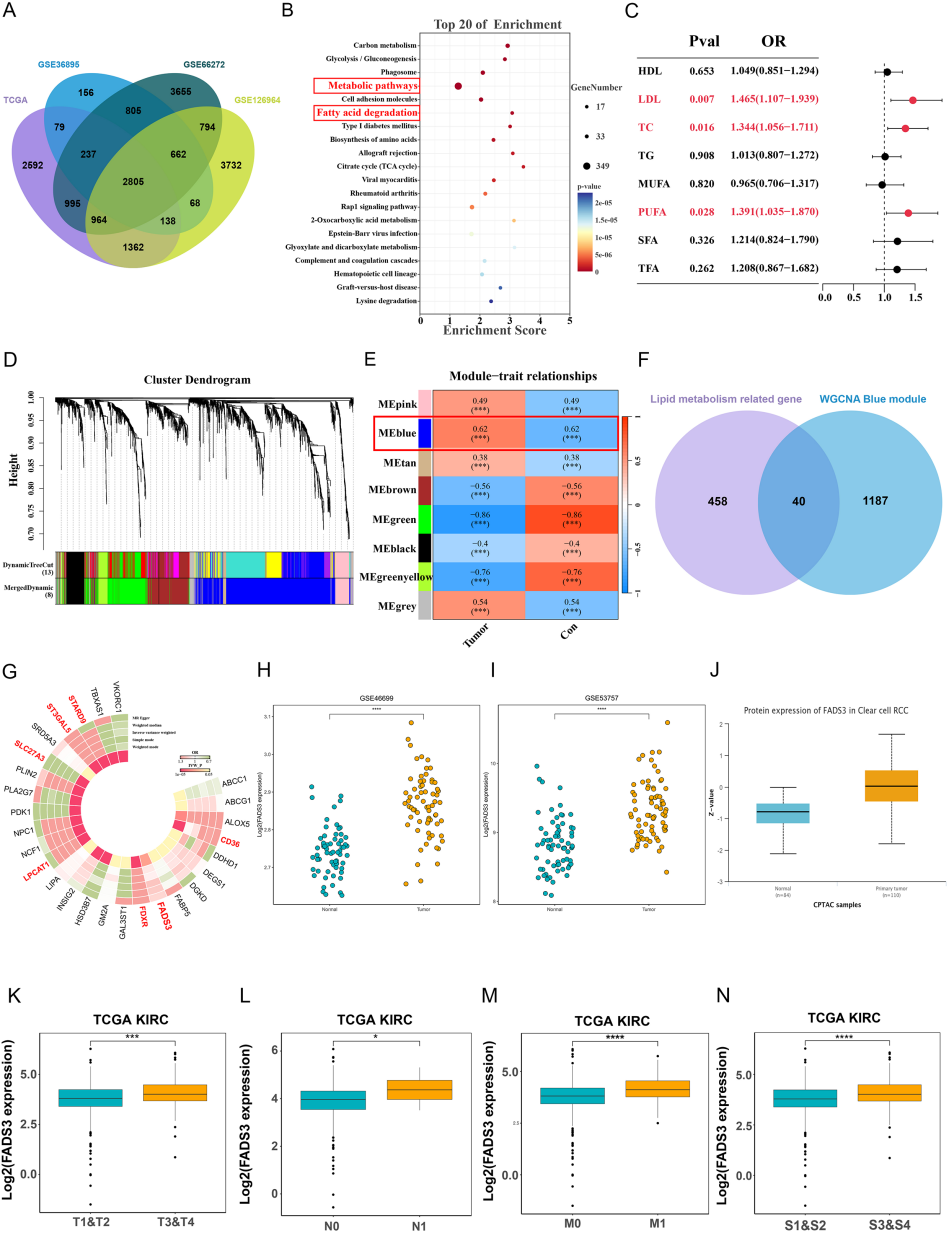
FADS3 as the core lipid metabolism-related gene in ccRCC. **(A)** Venn diagram of DEGs shared among four bulk transcriptome datasets. **(B)** GO functional enrichment analysis of the intersecting genes identified from the overlap analysis. Dot size denotes the number of enriched genes and dot color reflects statistical significance (adjusted *P* value). **(C)** Forest plot of univariable MR estimates for the effects of circulating lipid traits on ccRCC risk. **(D)** Dendrogram of topological overlap among clusters of DEGs. **(E)** Heatmap of module–trait relationships between WGCNA modules and tumor/normal status. **(F)** Venn diagram showing the overlap between lipid metabolism–related genes and genes in the WGCNA blue module. **(G)** Circular heatmap depicting the association patterns of the 40 candidate genes across multiple lipid traits, with colors indicating the direction and magnitude of the effects. **(H, I)** FADS3 mRNA expression in normal and tumor tissues from different datasets: GSE46699 **(H)**, and GSE53757 **(I)**. **(J)** Protein expression of FADS3 in ccRCC samples from CPTAC. **(K–N)** Boxplots illustrating FADS3 expression across various clinical factors in TCGA KIRC cohort. **(K)** Tumor stage (T1&T2 vs. T3&T4), **(L)** Tumor stage (N0 vs. N1), **(M)** Tumor stage (M0 vs. M1), **(N)** Subtypes (S1&S2 vs. S3&S4). *P < 0.05; ***P < 0.001; ****P < 0.0001.

Transcriptomic profiles of ccRCC tumor tissues were compared with those of adjacent normal tissues. DEGs identified in four transcriptomic datasets were intersected to generate a consensus DEG set (**Figure 1A**). Subsequent enrichment analysis of these DEGs showed significant enrichment in pathways including lipid metabolism (**Figure 1B**). MR analysis was used to provide genetically anchored evidence supporting potential causal associations between lipid metabolism-related exposures and ccRCC risk. The results indicated that LDL, TC, and PUFAs played a significant role in promoting ccRCC (**Figure 1C**). WGCNA analysis on the DEGs revealed the blue module showed the strongest promoting effect on ccRCC (**Figures 1D, E**). Lipid metabolism-related genes from Lipid Maps and GeneCards were intersected with the genes from the blue module, yielding 40 lipid metabolism-related genes in ccRCC (**Figure 1F**). eQTL-based MR analysis was performed for validation and seven core lipid-metabolism-related genes remained after pleiotropy exclusion (**Figure 1G**). Among these candidates, FADS3 emerged as a gene of particular interest, as it represents a novel gene in ccRCC that has not been thoroughly investigated.

To investigate FADS3 expression in ccRCC, we used array RNA sequencing profiles from the GEO expression profiles, containing ccRCC tumor and non-tumor specimens. The results showed that FADS3 gene expression levels in ccRCC samples were significantly elevated in these two cohorts from GEO databases (**Figures 1H, I**). Simultaneously, we verified using the CPTAC database that FADS3 protein expression was significantly elevated in ccRCC samples (**Figure 1J**). To investigate the clinical prognosis of high FADS3 expression in the ccRCC cohort, we first divided patients equally into two groups (FADS3-high and FADS3-low) based on the median FADS3 expression level (**Figures 1K–N**). Results indicated that significantly high FADS3 expression was clinically associated with high pathological grade (T, N, M stages) and clinical stage.

**Figure 2:**
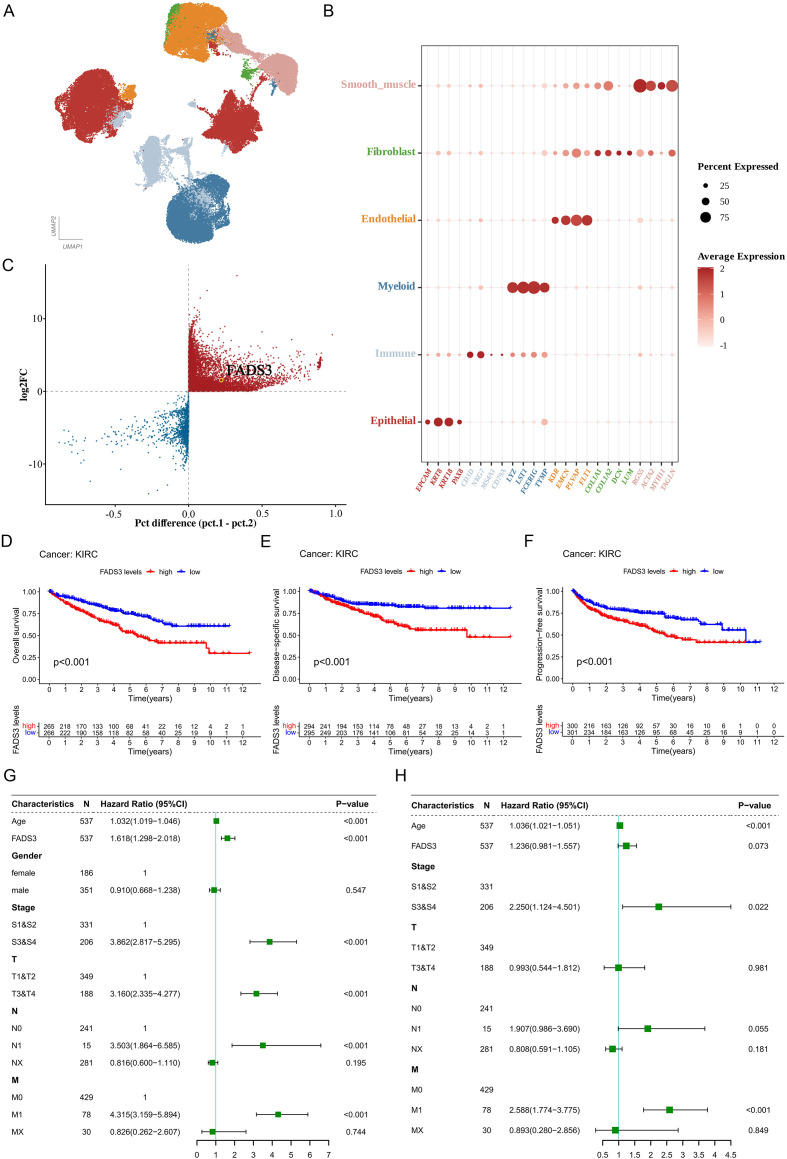
Upregulation of FADS3 in tumor samples predicts poor prognosis in ccRCC. **(A)** UMAP plot of the integrated single-cell RNA-sequencing data from GSE156632 and GSE159115, showing the major cell populations in the ccRCC microenvironment. **(B)** Dot plot of canonical marker genes across the major cell populations in the integrated ccRCC single-cell dataset. Dot size indicates the percentage of expressing cells, and color indicates the average expression level. **(C)** Differential gene expression between malignant and non-malignant epithelial cells in the integrated ccRCC single-cell dataset. FADS3 is highlighted. **(D–F)** Kaplan-Meier survival curves for overall survival **(D)**, disease-free survival **(E)**, and progression-free survival **(F)** in ccRCC patients from TCGA KIRC, stratified by FADS3 expression levels (high vs. low). **(G, H)** Forest plots showing univariate **(G)** and multivariate **(H)** Cox regression analyses for the association between FADS3 expression and survival outcomes in ccRCC patients. In **(G)**, FADS3 is an independent prognostic factor for overall survival (Hazard Ratio [HR] = 1.61, *p* < 0.001). In **(H)**, FADS3 shows a trend towards significance for progression-free survival (HR = 1.24, *p* = 0.073).

To explore FADS3 expression across tumor-microenvironment cell subsets, we analyzed a publicly available single-cell RNA-sequencing dataset. After integration of the GSE156632 and GSE159115 single-cell transcriptomic datasets, UMAP dimensionality reduction revealed that the ccRCC microenvironment was mainly composed of epithelial cells, immune cells, myeloid cells, endothelial cells, fibroblasts, and smooth muscle cells. The marker gene dot plot showed that each cell population highly expressed its corresponding canonical markers. For example, epithelial cells highly expressed EPCAM, KRT8, KRT18, and PAX8; myeloid cells highly expressed LYZ, LST1, and FCER1G; and endothelial cells highly expressed KDR and other vascular-related markers, supporting the reliability of the cell annotation (**Figures 2A, B**). Further inferCNV analysis of epithelial cells revealed prominent chromosomal copy number alteration patterns in tumor epithelial cells, whereas normal epithelial cells exhibited relatively stable CNV signals. Based on the high-confidence malignant and non-malignant clusters identified by inferCNV, differential expression analysis was subsequently performed. The results demonstrated marked transcriptional differences between malignant and non-malignant epithelial cells, and the candidate gene FADS3 showed an upregulated trend in malignant epithelial cells, suggesting that it may be involved in the molecular regulation of malignant epithelial cells in ccRCC. (**Figure 2C**).

### 3.2 FADS3 upregulation in ccRCC tumors predicts poor prognosis

Comprehensive survival analyses of ccRCC patients were performed to delineate the association between FADS3 expression and tumor prognosis. Survival analysis revealed that the Overall Survival (OS), Progression-Free Survival (PFS), and Disease-Specific Survival (DSS) rates of patients in the FADS3-high group were significantly lower than those in the FADS3-low group (**Figures 2D–F**). Univariate Cox regression analysis showed that high FADS3 expression was significantly associated with poor prognosis in ccRCC patients (**Figure 2G**). However, in multivariate regression including factors such as age, stage, and grade, it was no longer statistically significant (**Figure 2H**). This may be related to the high correlation between FADS3 and tumor stage/grade.

**Figure 3:**
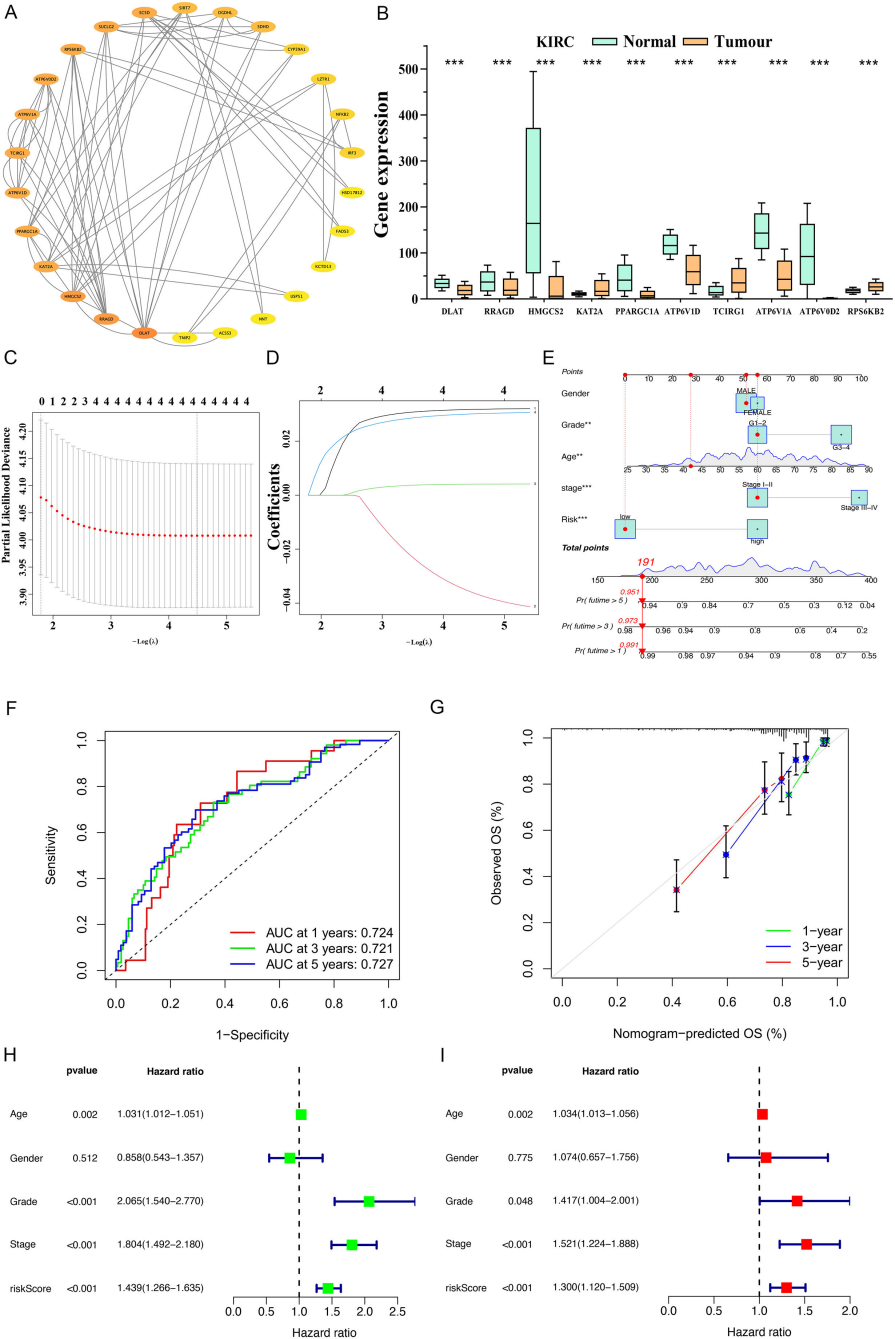
Machine learning-based construction of ccRCC prognostic model. **(A)** Selection of 50 genes positively and 50 genes negatively correlated with FADS3. The top 10 hub genes with the highest connectivity degree were identified from the PPI network. **(B)** Box plots illustrating the differential expression levels of The top 10 hub genes of the PPI network in **(A)**. **(C, D)** Screening of prognostic genes using LASSO Cox regression analysis. **(C)** Cross-validation for tuning parameter selection. **(D)** LASSO coefficient profiles of the hub genes. **(E)** A predictive nomogram integrating the risk score with clinicopathological features (gender, grade, age, and stage) to predict the 1-, 3-, and 5-year overall survival (OS) probability. **(F)** Time-dependent ROC curves for evaluating the predictive performance of the risk signature for 1-, 3-, and 5-year OS. **(G)** Calibration curves showing the concordance between the nomogram-predicted and observed survival rates at 1, 3, and 5 years. **(H, I)** Assessment of independent prognostic factors. **(H)** Univariate Cox regression analysis and **(I)** Multivariate Cox regression analysis of the risk score and clinical characteristics. HR, hazard ratio. ***P < 0.001.

Analysis of genes co-expressed with FADS3 in ccRCC provided valuable insights into potential regulatory networks and prognostic model. Through the cBioPortal online analysis website (https://www.cbioportal.org/), we identified the top 50 genes positively and negatively correlated with FADS3. We performed PPI network analysis on the proteins encoded by these 50 positively and 50 negatively correlated genes using the STRING database and Cytoscape (**Figure 3A**), and identified the top 10 hub genes (DLAT, RRAGD, HMGCS2, KAT2A, PPARGC1A, ATP6V1D, TCIRG1, ATP6V1A, ATP6V0D2, RPS6KB2) using the CytoHubba plugin (**Figure 3B**). These 10 hub genes highlight potential central regulators in the biological context of FADS3-related ccRCC. Combining these hub genes with FADS3 may improve the accuracy of prognosis prediction for ccRCC patients.

Univariate Cox regression analysis (*p* < 0.05) was used to screen key genes significantly associated with patient prognosis. Subsequently, Lasso regression was applied for feature selection (**Figures 3C, D**). Based on the retained candidate genes, multivariate Cox regression analysis was further employed to determine independent prognostic factors, and a risk score model was constructed based on regression coefficients: RiskScore = (FADS3 Expression * 0.0368) - (DLAT Expression * 0.0462) + (TCIRG1 Expression * 0.0323). A Nomogram was constructed using the RiskScore, integrating clinical pathological characteristics and the prognostic risk score to visually predict the 1-year, 3-year, and 5-year overall survival rates of KIRC patients (**Figure 3E**). Finally, using the median risk score as the cutoff, patients were divided into high-risk and low-risk groups, then time-dependent ROC curves were plotted to quantitatively evaluate the model’s efficacy in predicting 1-year, 3-year, and 5-year overall survival (OS) of patients (**Figure 3F**), and accuracy was judged via the area under the curve (AUC) and the calibration curves (**Figures 3F, G**). We further analyzed the relationship between clinical pathological characteristics of KIRC patients and the prognostic model via univariate and multivariate Cox regression (**Figures 3H, I**). The machine learning-based prognostic model indicated a more significant role of the FADS3 in ccRCC prognosis.

### 3.3 FADS3 upregulation in ccRCC affects immune cell infiltration

**Figure 4:**
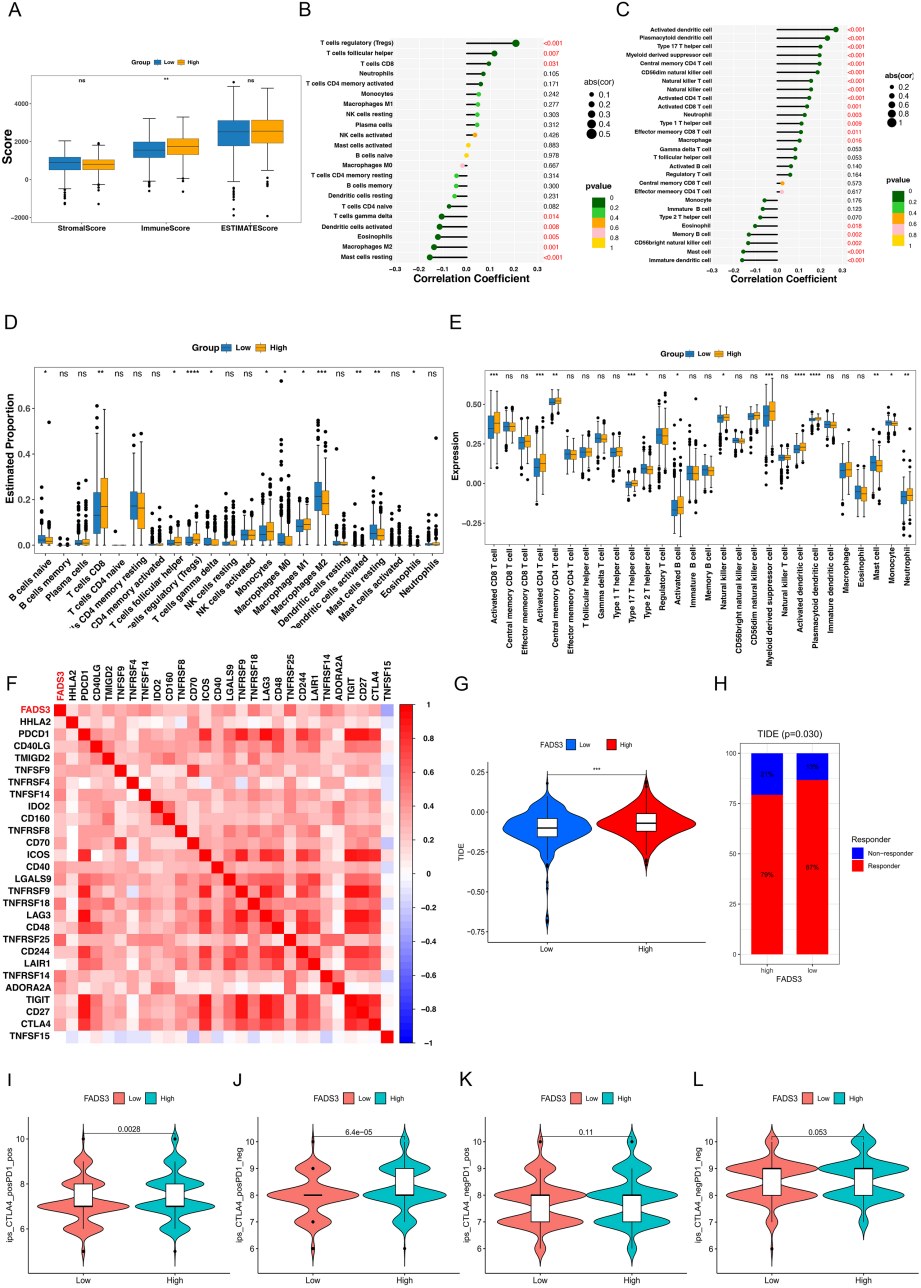
FADS3 association with immune infiltration and immunotherapeutic response in ccRCC. **(A)** Boxplots showing immune scores, stromal scores, and ESTIMATE scores across ccRCC samples with high and low FADS3 expression. Significant differences are observed in immune scores (*p* < 0.01) between high and low FADS3 groups. **(B, C)** Correlation between FADS3 expression and immune cell infiltration across multiple immune cell types in the ccRCC dataset. **(B)** The correlation of FADS3 with regulatory T cells, CD8+ T cells, macrophages, and other immune cells. **(C)** The correlation of FADS3 with dendritic cells, natural killer **(NK)** cells, monocytes, and macrophage polarization. Significant correlations with immune infiltration were observed (*p* < 0.01). **(D)** Estimated immune cell proportions across low and high FADS3 expression groups, showing differences in B cells, T cells, macrophages, and neutrophils. **(E)** Expression of immune-related genes across low and high FADS3 expression groups. The figure highlights differences in the expression of genes related to immune cell infiltration, with significant changes in certain gene expressions. **(F)** A heatmap showing the correlation matrix between FADS3 expression and immune checkpoints, cytokines, and other immune-related markers. **(G)** Violin plot showing the Tumor Immune Dysfunction and Exclusion (TIDE) scores between the low and high FADS3 expression groups. **(H)** Bar chart illustrating the proportion of responders and non-responders to immunotherapy based on FADS3 expression levels. **(I–L)** TCIA analysis boxplots showing therapeutic outcomes in high versus low FADS3 expression groups treated with anti-CTLA4 monotherapy or combination anti-CTLA4 and anti-PD1 therapy. *P < 0.05; **P < 0.01; ***P < 0.001; ****P < 0.0001;"ns" stands for "not significant".

To reveal the complex relationship between FADS3 expression and the tumor immune response in ccRCC, we carefully analyzed the immune microenvironment. Since TME scores reflect the abundance of immune and stromal factors, we utilized the ESTIMATE algorithm. Results indicated a higher Immune Score in the FADS3-high group (**Figure 4A**). Next, to explore the composition of 22 immune cell types in the TME under different FADS3 levels, the CIBERSORT algorithm was applied. Results showed that the proportions of regulatory T (Treg) cells, T follicular helper (TFH) cells, and CD8^+^ T cells were highest in the FADS3-high group, whereas those of resting mast cells, M2 macrophages, and eosinophils were lowest (**Figure 4B**). The ssGSEA algorithm predicted that the FADS3-high group exhibited higher amounts of myeloid-derived suppressor cells, activated dendritic cells, Th17 cells, and others (**Figure 4C**). These results suggest that FADS3 up-regulation is associated with an altered and likely functionally dysregulated TIME in ccRCC.

To further assess the relationship between FADS3 and immune infiltration, we used ssGSEA and CIBERSORT as complementary analyses. In CIBERSORT, high FADS3 expression was positively correlated with regulatory T cells (Tregs), T follicular helper (TFH) cells, and CD8+ T cells, and negatively correlated with resting mast cells, M2 macrophages, eosinophils, activated dendritic cells, and naïve CD4+ T cells (**Figure 4D**). In ssGSEA, high FADS3 expression was positively associated with activated DCs, Th17 cells, NK cells, and activated CD4+/CD8+ T cells, but negatively associated with immature DCs, mast cells, eosinophils, and several B-cell subsets (**Figure 4E**). Collectively, these findings indicate that high FADS3 expression is associated with a markedly altered immune contexture in ccRCC. Rather than simply reflecting a uniformly activated antitumor immune state, this pattern is more consistent with an immune-infiltrated but functionally dysregulated microenvironment, characterized by the coexistence of infiltrating effector-associated cells and suppressive or dysfunction-related immune features. Differences between algorithms may reflect distinct gene-set definitions and deconvolution strategies, and the biological significance of these findings warrants further experimental validation.

### 3.4 FADS3 upregulation in ccRCC affects immunotherapy response

We systematically analyzed the correlation between FADS3 and immune checkpoint molecules. Results showed FADS3 was significantly positively correlated with multiple immune regulatory molecules, including TNFRSF25, TNFRSF14, TNFRSF18, TNFSF14, LGALS9, and CD40 (r = 0.39–0.59, *p* < 1×10^-20^), suggesting a key role in TME regulation (**Figure 4F**). FADS3 also showed moderate positive correlation with typical checkpoints like PD-1 (PDCD1) and CTLA-4 (r ≈ 0.33, *p* < 1×10^-15), supporting a link to immune suppression and T cell exhaustion. Notably, FADS3 was negatively correlated with molecules such as TNFSF15 (r = -0.33) and PD-L1 (CD274) (r = -0.11), indicating a complex, dual mechanism affecting immune balance. This suggests FADS3 may not only serve as a potential biomarker reflecting tumor immune status but also provides evidence for its role in the interaction between lipid metabolism and immune checkpoints.

To assess potential benefits of ICI treatment in different FADS3 expression groups, we used TIDE scores. The FADS3-high group had higher TIDE scores than the FADS3-low group, suggesting poorer immunotherapy efficacy and lower response rates (**Figure 4G**). Furthermore, TCIA analysis assessed the relationship between FADS3 expression and PD-1/CTLA-4 drug efficacy. Results indicated that for combined anti-CTLA-4 and anti-PD-1 treatment, or anti-CTLA-4 treatment alone, the FADS3-high group might have better efficacy (**Figures 4H–L**). In summary, FADS3 expression may be related to the efficacy of immunotherapy drugs, representing another potential factor affecting ccRCC prognosis.

### 3.5 FADS3 knockdown compromises malignant phenotypes of ccRCC cells

**Figure 5:**
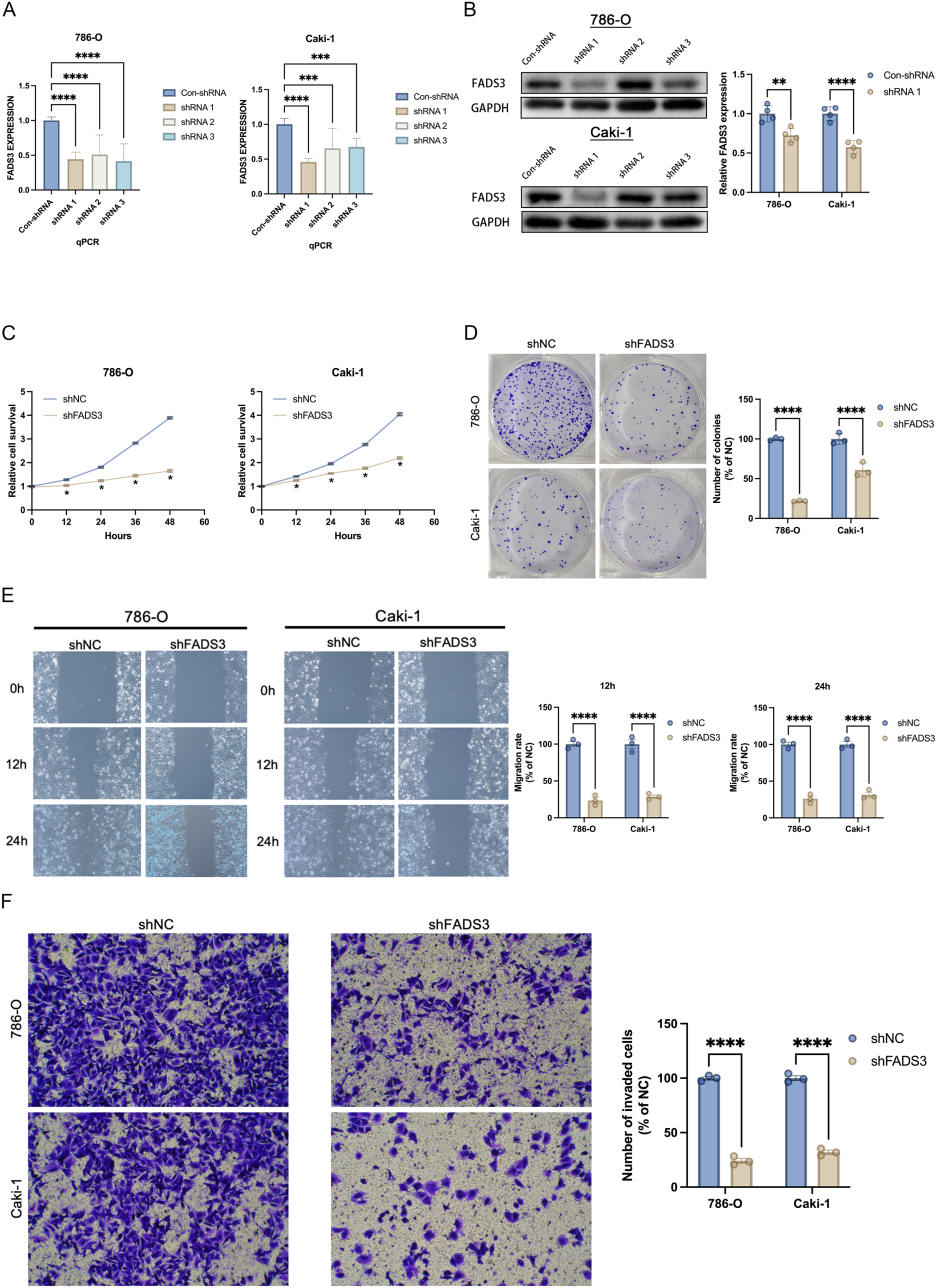
FADS3 knockdown-induced attenuation of malignant biological behaviors in ccRCC cells. **(A)** Quantitative PCR (qPCR) analysis showing the knockdown efficiency of FADS3 in 786-O and Caki-1 cells after transfection with three different shRNAs (shRNA1, shRNA2, shRNA3) compared to control shRNA (shNC). **(B)** Western blot analysis confirming the knockdown of FADS3 protein expression in 786-O and Caki-1 cells. GAPDH was used as a loading control. **(C)** Cell proliferation assay showing the relative cell survival rate of 786-O and Caki-1 cells at different time points after FADS3 knockdown. **(D)** Colony formation assay showing the number of colonies in 786-O and Caki-1 cells transfected with shNC (Con-shRNA) or shFADS3 (shRNA1). Quantification is shown as the percentage of colonies relative to the control group. **(E)** Wound healing assay showing the migration rate of 786-O and Caki-1 cells after scratching. Migration rate is quantified as the percentage of wound closure compared to the control group. **(F)** Transwell invasion assay showing the number of invaded 786-O and Caki-1 cells after FADS3 knockdown. Quantification is shown as the percentage of invaded cells relative to the control group. Data are presented as the mean ± SD (n = 3-4). *P <0.05; **P <0.01; ***P <0.001; ****P <0.0001.

To confirm the tumorigenic roles of FADS3, the malignant phenotypes of ccRCC cell lines were evaluated using a panel of functional assays. Stable ccRCC cell lines harboring lentiviral shRNA-mediated FADS3 knockdown or control scramble shRNA were established. Knockdown efficiency was confirmed by RT-qPCR and Western blotting (**Figures 5A, B**). In two ccRCC cell lines, 786-O and Caki-1 cells, FADS3 silencing markedly impaired proliferative capacity, as determined by cell-viability assays (**Figure 5C**). Consistently, colony-formation assays revealed that FADS3 depletion significantly suppressed the clonogenic potential of both cell lines (**Figure 5D**). Wound-healing assays demonstrated that FADS3 down-regulation substantially attenuated the migratory capacity of each ccRCC line (**Figure 5E**). Moreover, transwell invasion assays showed that FADS3 knockdown markedly reduced the invasive properties of the tumor cells (**Figure 5F**). Collectively, these data provide direct evidence that FADS3 expression is essential for the malignant phenotypes of ccRCC cells, aligning with its clinical significance in tumor development and progression.

### 3.6 FADS3 is associated with metabolic and oncogenic signaling pathways

**Figure 6:**
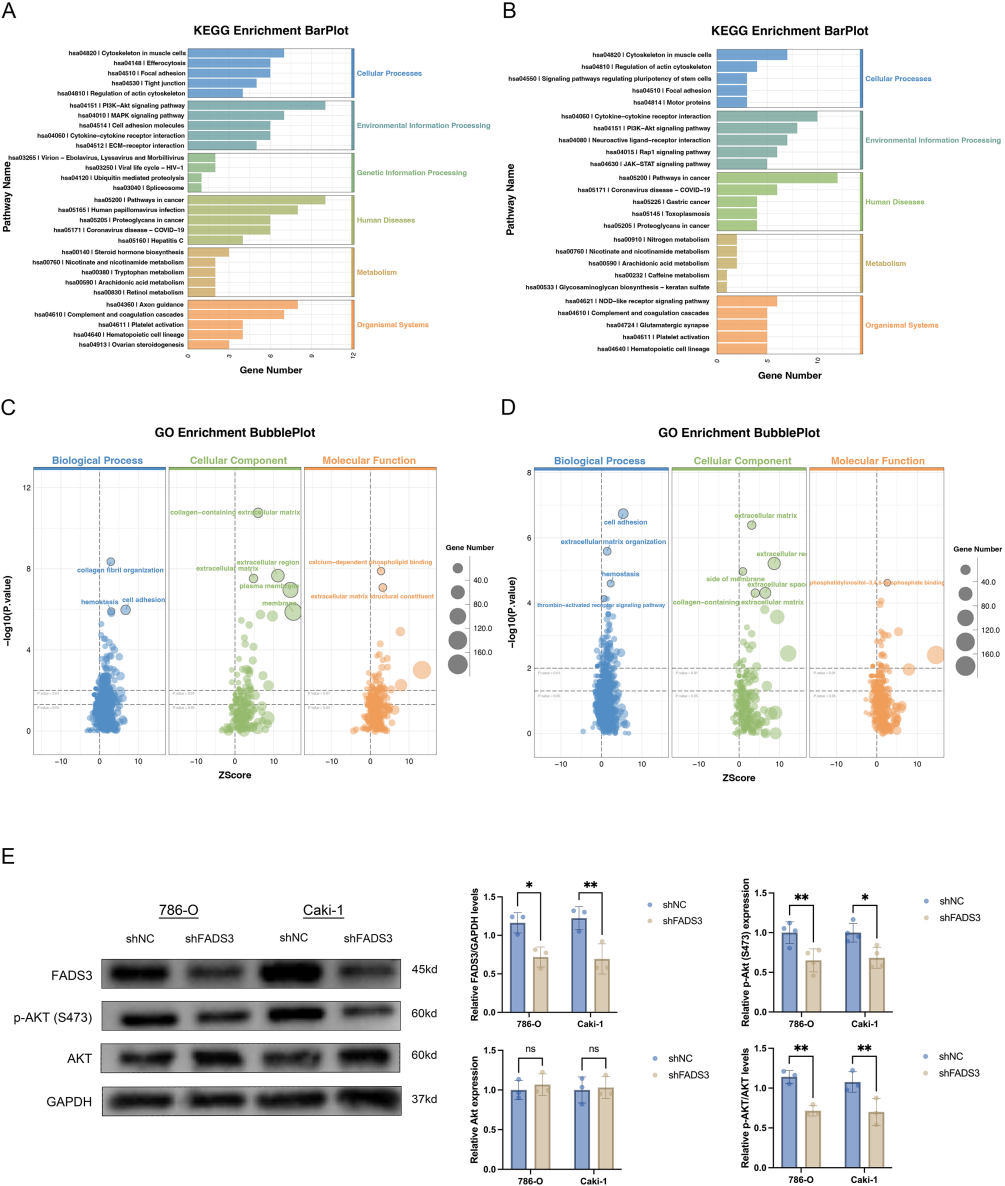
Functional enrichment analysis and immunoblotting validation of FADS3-mediated signaling pathways in ccRCC cells. **(A-B)** KEGG enrichment bar plot showing the top pathways significantly enriched in the genes differentially expressed after FADS3 knockdown in Caki-1 **(A)** and 786-O **(B)** cells. Pathways are grouped by biological processes such as Cellular Processes, Environmental Information Processing and Metabolism. **(C-D)** GO enrichment bubble plot representing the top biological processes, cellular components, and molecular functions enriched in the FADS3 knockdown gene list. The size of the bubbles corresponds to the number of genes in each category, and the color indicates the statistical significance of the enrichment (log10 *p*-value). **(E)** Western blot analysis showing the expression of FADS3, phospho-Akt (S473), and total Akt in the Caki-1 and 786-O cells after FADS3 knockdown. Data are presented as the mean ± SD (n = 3-4). ns, not significant; *P < 0.05; **P < 0.01.

To elucidate the molecular mechanisms underlying the tumorigenic roles of FADS3, RNA-sequencing was performed on ccRCC cells with or without FADS3 knockdown. Differential expression analysis identified a substantial set of overlapping genes altered in both 786-O and Caki-1 cells (**Figures 6A, B**), indicating that FADS3 downregulation elicits consistent transcriptional effects across different ccRCC cell lines. KEGG pathway enrichment analysis demonstrated that DEGs were significantly enriched in pathways associated with cellular metabolism and tumor progression (**Figures 6A, B**).

Gene Ontology (GO) enrichment analysis (**Figures 6C, D**) revealed profound alterations across three major categories. Regarding Biological Processes (BP), terms such as fatty acid metabolic process, lipid catabolic process, and the regulation of cell migration and motility were highly enriched. In terms of Cellular Components (CC), DEGs were primarily localized to plasma membrane microdomains (lipid rafts), the mitochondrial matrix, and peroxisomes. These associations strongly support the involvement of FADS3 in membrane lipid remodeling and the regulation of metabolic organelles. Regarding Molecular Function (MF), significant enrichment was observed in oxidoreductase activity, lipid binding, and kinase regulator activity. These functional profiles align with the observed impairment of lipid desaturation and downstream signal transduction in FADS3-downregulated cells.

The most prominently enriched signaling pathway identified by KEGG analysis was the PI3K/Akt signaling pathway, indicating that this pathway is robustly regulated by FADS3 (**Figures 6A, B**). To validate this observation, Western blotting was employed to evaluate the phosphorylation status of Akt at Ser473, a well-established indicator of PI3K/Akt pathway activation. FADS3 knockdown significantly reduced the relative phosphorylation levels of Akt at Ser473, indicating diminished pathway activation (**Figure 6E**). These findings demonstrate that FADS3 promotes ccRCC progression, at least in part, by maintaining the active state of the PI3K/Akt signaling cascade.

**Figure 7:**
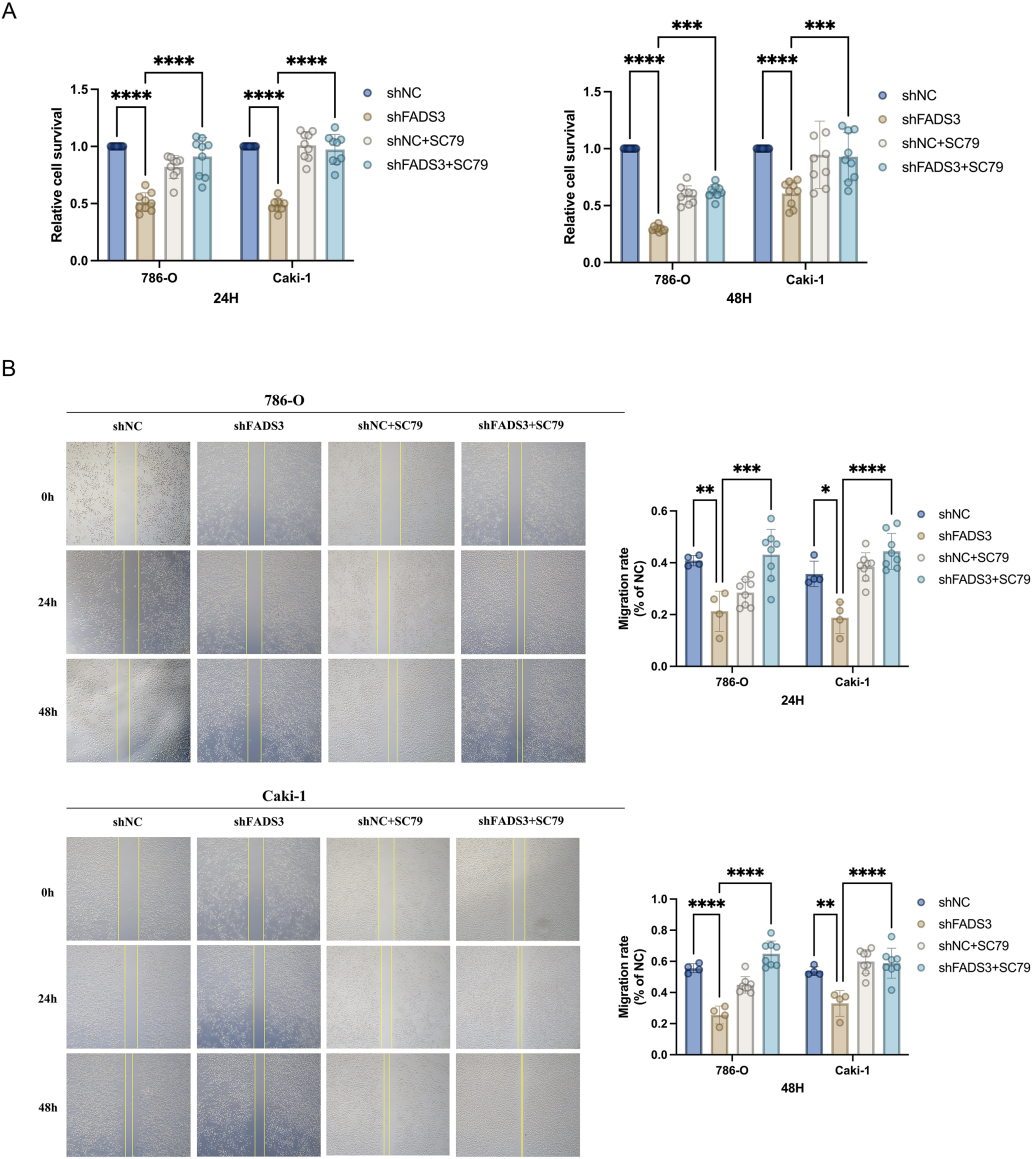
Effects of Akt activation on FADS3-knockdown-mediated inhibition of ccRCC cell proliferation and migration. **(A)** Cell proliferation assay. FADS3-knockdown or wild-type 786-O and Caki-1 cells were treated with or without the Akt activator SC79 (10 µM). Relative cell proliferation was measured at 24 and 48 hours by MTT assay. **(B)** Cell migration assay. Wound-healing assays were performed to evaluate the migratory capacity of FADS3-knockdown or wild-type 786-O and Caki-1 cells following treatment with or without SC79 (10 µM) at 24 and 48 hours. Data are presented as the mean ± SD (n = 3-4). ns, not significant; *P < 0.05; **P < 0.01. ***P < 0.001; ****P < 0.0001.

To further verify the functional involvement of PI3K/Akt signaling, rescue assays were conducted using the AKT activator SC79 (10 µM). SC79 treatment partially reversed the inhibitory effects of FADS3 knockdown on cell survival and migration in both 786-O and Caki-1 cells, as demonstrated by MTT and wound-healing assays at 24 and 48 h (**Figures 7A, B**). These results further indicate that FADS3 exerts its pro-proliferative and pro-migratory effects, at least in part, through PI3K/Akt pathway activation.

### 3.7 FADS3 expression in patient ccRCC tumors affects immune cell behavior

**Figure 8:**
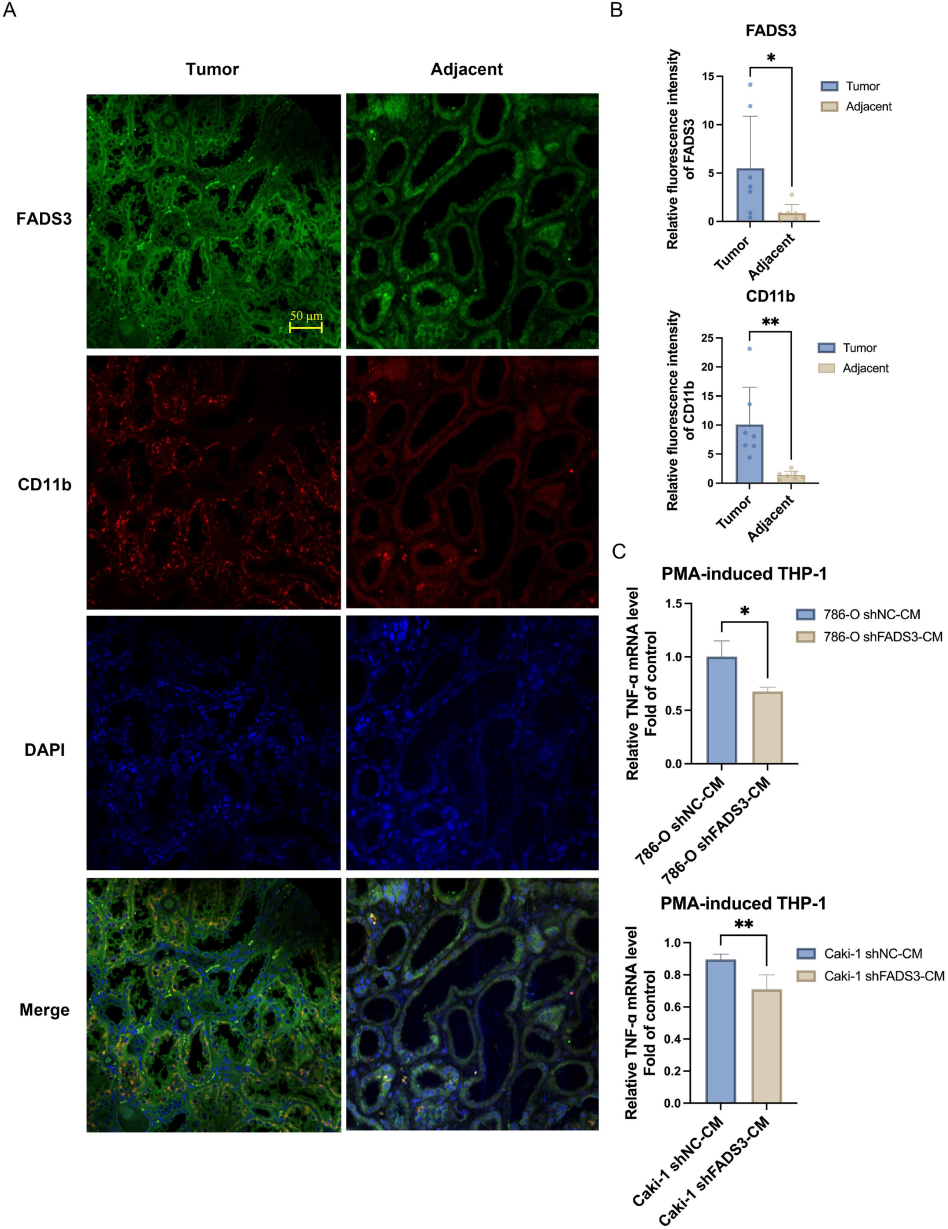
FADS3 expression in ccRCC tumor tissues and its relationship with immune cell behavior. **(A)** Multiplex immunofluorescence staining of FADS3 andCD11b in ccRCC tumor and adjacent tissues. Representative images of FADS3 (green) and CD11b (red) fluorescence intensities. Nuclei werecounterstained with DAPI (blue). Scale bar, 50 µm. **(B)** Quantitative analysis of FADS3 and CD11b expression in ccRCC tumor tissues. **(C)** Effects of FADS3 knockdown in ccRCC cells on TNF-a expression in macrophages. Conditioned medium from shFADS3-transduced ccRCC cells reducedTNF-a expression in PMA-differentiated THP-1 cells. PMA-differentiated THP-1 cells were stimulated with conditioned medium from shNC- orshFADS3-transduced 786-O and Caki-1 cells, and TNF-a mRNA levels were quantified by qRT-PCR. Data are presented as the mean ± SD (n = 3-4). *P < 0.05; **P < 0.01.

FADS3 expression in ccRCC was further validated by immunofluorescence staining of patient tumor samples. The analysis was conducted using a ccRCC tissue microarray, wherein tumor samples were immunostained with FADS3-specific antibodies and corresponding fluorescent secondary antibodies. Higher FADS3 expression was observed in ccRCC tumor tissues compared with adjacent normal tissues (**Figure 8A**). Concurrently, the TAM marker CD11b was also immunostained in these tumors. ccRCC tumor samples exhibited stronger CD11b staining than adjacent tissues, indicating increased TAM infiltration in tumor tissues relative to normal tissues (**Figure 8A**). These findings suggest that elevated FADS3 expression in ccRCC is associated with enhanced CD11b-positive myeloid cell infiltration (**Figure 8B**).

To further investigate how FADS3 expression in tumor cells affects TAM function, conditioned medium from ccRCC cells was used to treat macrophages *in vitro*. THP-1 cells were differentiated into macrophages and stimulated with conditioned medium from FADS3-knockdown or wild-type 786-O or Caki-1 cells. FADS3 knockdown significantly decreased TNF-α mRNA expression in PMA-differentiated THP-1 cells compared with conditioned medium from wild-type tumor cells (**Figure 8C**). These results suggest that FADS3 expression in ccRCC tumors is required for tumor cell-derived paracrine signaling and TAM functional polarization.

## 4 Discussion

ccRCC is the most common and aggressive subtype of RCC, representing a significant cause of cancer-related mortality worldwide (4). Although therapeutic strategies with ICIs have achieved remarkable progress, a considerable proportion of patients still fail to achieve durable responses or eventually develop resistance (34). There is an urgent need to identify novel molecular targets and therapeutic strategies to complement existing treatments. In this study, we conducted comprehensive bioinformatic analyses and experimental validations to reveal that FADS3 promotes ccRCC progression, alters the TIME and immunotherapy responses, and correlates with poor prognosis.

FADS3 belongs to the fatty acid desaturase (FADS) gene family, within which FADS1 and FADS2 have been clearly demonstrated to participate in the biosynthesis of PUFAs (24). In contrast, FADS3 has long been regarded as an atypical desaturase, with ongoing debates regarding its substrate specificity and molecular function (35). Recent studies suggest that FADS3 may primarily function as a sphingoid Δ14Z desaturase, regulating sphingolipid metabolism, rather than directly involving in the classical PUFA synthesis pathway (36). Sphingolipids, such as ceramide and sphingosine-1-phosphate, are a class of important bioactive lipids that play critical roles in cancer cell proliferation, apoptosis, migration, and immune regulation (37). Accumulating evidence indicates that aberrant sphingolipid metabolism is closely associated with the progression and immune evasion of various tumors (38). However, the relationship between FADS3 and tumorigenesis, particularly its role in ccRCC, remains insufficiently explored. Given the high dependence of ccRCC on lipid remodeling and membrane lipid homeostasis, along with the observation that FADS3 is highly expressed exclusively in ccRCC compared to other urological tumors, we speculate that FADS3 may play a more significant role in ccRCC by substituting for other desaturases or by influencing tumorigenesis through sphingolipid metabolism.

In this study, we demonstrated that FADS3 is significantly upregulated in ccRCC tumors and is closely associated with advanced tumor stage and poor prognosis. FADS3 upregulation is also linked to altered immune cell infiltration and immunotherapy response. These findings reflect FADS3-mediated reprogramming of lipid metabolism and the metabolic plasticity of ccRCC tumors, which may arise from epigenetic modifications and other intracellular changes during tumorigenesis, as suggested by previous studies (39). Our immune-related analyses suggest that FADS3-high ccRCC does not simply represent an immune-desert phenotype. Instead, this subgroup appears to exhibit an immune-infiltrated but functionally dysregulated state. On the one hand, high FADS3 expression was associated with increased immune scores and enrichment of several infiltrating immune-cell subsets. On the other hand, the FADS3-high group also displayed increased TIDE scores and positive correlations with multiple immune checkpoint-related molecules, supporting the presence of immune dysfunction, exhaustion-related features, or immune escape. This framework may help explain why increased immune-cell infiltration, including CD8+ T-cell-associated signals, does not necessarily translate into improved predicted response to immunotherapy. In this context, FADS3 may be relevant not only as a prognostic biomarker but also as a marker of metabolically linked immune dysregulation in ccRCC.

Our findings on FADS3 align with observations that FADS7/8 expression is elevated in breast cancer patients with high lymph node metastasis (40), suggesting FADS family enzymes may regulate immune cell recruitment and function through PUFA metabolism. Fatty acid metabolism in TAMs promotes fatty acid oxidation and IRF pathway activation, driving CXCL10/CXCL11 secretion and subsequent CD8^+^ T cell infiltration (41). As a key enzyme in PUFA biosynthesis, FADS3 likely modulates TAM polarization and immunomodulatory capacity through lipid mediator production. Aberrant lipid metabolism further contributes to immune dysfunction, including T cell exhaustion and M2 macrophage polarization, fostering an immunosuppressive microenvironment (42, 43). Importantly, targeting lipid metabolic nodes such as FASN and FABP5 has proven effective in reversing immunosuppression and enhancing anti-tumor immunity (44, 45). This supports exploring FADS3 as a therapeutic target to rewire metabolic-immune crosstalk and restore productive immune surveillance.

Our *in vitro* functional assays revealed that FADS3 knockdown markedly inhibited the proliferation, migration, and invasion capabilities of ccRCC cells. Transcriptomic analysis indicated significant remodeling of multiple metabolic pathways and oncogenic signaling cascades, with particularly prominent inhibition of the PI3K/Akt signaling pathway. Notably, phosphorylation levels of AKT were substantially reduced after FADS3 knockdown, while total Akt expression remained largely unchanged, suggesting that FADS3 likely promotes tumor progression by sustaining Akt activation rather than by regulating its expression level. Previous studies have shown that fatty acid desaturation and cellular membrane lipid composition critically influence PI3K/Akt signal transduction (46–49). Therefore, it is conceivable that FADS3 may facilitate the malignant progression of ccRCC via modulation of the PI3K/Akt pathway. Beyond its role in tumor cells, PI3K/Akt signaling is also an important regulator of immune-cell function, particularly in T-cell responses, immune checkpoint-associated signaling, and myeloid/macrophage behavior (50, 51). Therefore, the immune alterations observed in the FADS3-high subgroup may, at least in part, be related to PI3K/Akt-associated immune remodeling. However, the current study experimentally validated reduced Akt phosphorylation only in ccRCC tumor cells following FADS3 knockdown. Whether FADS3 directly regulates PI3K/Akt signaling in immune cells remains unresolved. Furthermore, the exact roles of FADS3 and PI3K/Akt signaling in ccRCC will require further investigation, particularly through *in vivo* studies.

In summary, this study systematically reveals the important role of FADS3 as a lipid metabolism-related oncogenic factor in ccRCC. By integrating bioinformatic analysis with functional experiments, we demonstrate that FADS3 is involved in ccRCC progression, metabolic reprogramming, oncogenic signaling activation, and regulation of the TIME in ccRCC. Given the high expression of FADS3 in ccRCC compared to other urological tumors, future research should further elucidate the specific mechanisms by which FADS3 promotes ccRCC development, particularly determining whether it drives malignant progression through PUFA or sphingolipid desaturation. Additionally, developing FADS3 inhibitors and exploring their potential to exert therapeutic effects in ccRCC patients in clinical settings is warranted.

## Data Availability

The datasets presented in this study can be found in online repositories. The names of the repository/repositories and accession number(s) can be found in the article/**Supplementary Material**.
